# Towards a systematic evaluation of moral bioenhancement

**DOI:** 10.1007/s11017-022-09584-1

**Published:** 2022-07-24

**Authors:** Karolina Kudlek

**Affiliations:** 1 Department of Philosophy, University of Twente, Enschede, The Netherlands; 2 Institute of Philosophy, Zagreb, Croatia

**Keywords:** Moral bioenhancement, Moral neuroenhancement, Persson/Savulescu, Ethical guidelines, Moral permissibility

## Abstract

The ongoing debate about moral bioenhancement (MBE) has been exceptionally stimulating, but it is defined by extreme polarization and lack of consensus about any relevant aspect of MBE. This article reviews the discussion on MBE, showing that a lack of consensus about enhancements’ desirable features and the constant development of the debate calls for a more rigorous ethical analysis. I identify a list of factors that may be of crucial importance for illuminating the matters of moral permissibility in the MBE debate and which could help us move beyond the current lack of consensus. More precisely, I propose three important theoretical and normative standards that MBE should satisfy if we wish to mitigate the concerns about its utter impermissibility. Systematically assessing MBE interventions across the presented categories should provide valuable conclusions about its theoretical soundness and feasibility, its compatibility with fundamental moral norms, and its compatibility with or facilitation of socio-political goals of equality and justice.

## Introduction

Biomedical enhancements have been a subject of intense discussion over the past decade, with moral bioenhancement (MBE) perhaps causing even more scholarly disagreement than other forms of bioenhancement.^1^ Given its controversial and interdisciplinary nature, MBE is susceptible to various interpretations. For example, views on MBE range from advocating it be given priority over other modes of human enhancement because its role could be crucial for the successful continuation of human life [e.g., 1, 2], to the claims it goes against the very nature of morality and it should be downright rejected [e.g., 3–6]. Thus, although the ongoing debate has been exceptionally stimulating, it is defined by extreme polarization and lack of consensus about what moral enhancement is, whether we want it, and whether we should allow it.

In this article, I review the discussion about MBE, showing that a lack of consensus about enhancements’ desirable features and constant development of the debate call for a more rigorous ethical analysis. I identify a list of factors that may be of crucial importance for helping us understand what is or is not morally permissible, allowing us to move beyond the current lack of consensus. More precisely, I propose three important theoretical and normative standards that MBE should satisfy if we wish to, at least, mitigate the concerns about its utter impermissibility–if not even determine its moral status more clearly.^2^

These guidelines are influenced and shaped by the most pertinent concerns raised in the discussion because those concerns roughly indicate the requirements that an MBE proposal should ideally satisfy. These requirements include (i) plausible coherence, feasibility, and effectiveness of the enhancement project; (ii) the absence of conflict with fundamental moral values and norms; and (iii) compatibility with or facilitation of socio-political goals of equality and justice. This is not to say that meeting these requirements would guarantee the moral permissibility of MBE, but it would, at least, seriously challenge the common assumption about their outright impermissibility. Additionally, systematic examination and evaluation of an MBE project on these three levels should improve future discussions’ quality by providing valuable insight into a particular MBE project’s coherence, feasibility, and overall permissibility.

I proceed to summarize the current state of the debate by reviewing some of the most influential moral enhancement proposals (in the second and fourth section) and identifying three main categories of concerns raised explicitly in the context of MBE (in the third section). Finally, I introduce the above-described methodological framework and explain the purpose and application of the proposed requirements for a systematic evaluation of MBE’s moral permissibility.

## The need for moral bioenhancement

In their 2008 article, Persson and Savulescu argued that human enhancements, such as cognitive enhancement, present a threat unless we develop effective ways of improving humanity’s moral character [1]. They ground the urgent need for MBE on two main reasons. First, they make the case that increased scientific and technological progress can be dangerous for the survival of the human species. Second, they argue that our moral psychology is maladapted to our modern environment (which places us at an as-of-yet unprecedented risk of catastrophe). As a result, MBE should be purposed to compensate for the discrepancy between scientific and technological progress achieved by the human species over the past few centuries and the allegedly lagging progress in our species-typical moral psychology.

These drawbacks of our moral psychology are manifested in certain behavioral tendencies and biases that do not seem to be a good fit for the moral requirements of the modern world. For example, we are disposed to care much more about what happens in the near future and to those near and dear to us, than about the suffering of distant, unknown individuals and larger collectives [8, p. 338]. We are often inclined to morally undesirable behavior such as nepotism, xenophobia, and groupishness.^3^ Psychological myopia also causes inadequate responses in the context of care for future people, the environment, and non-human animals. On a more general level, one may conclude that outdated moral psychology mostly manifests as the absence of sufficient and adequate moral motivation.

Persson and Savulescu argue that our moral dispositions, such as altruism and a sense of justice, have biological foundations and could be modified by biomedical means (assuming that biomedicine has advanced to a sufficiently sophisticated state).^4^ More precisely, biomedical means could be used for reinforcing our altruism and making us more just or fair. This appears necessary because, as authors note, human beings will not by themselves spring into action necessary to avoid global collapse. Traditional moral enhancement, such as upbringing or education, and enhanced powers of reason are tremendously important, but they are insufficient when the span of necessary concern is rapidly expanding [2].

MBE seems to be largely a matter of motivating ourselves to do what we already know to be right–of overcoming our moral weakness of will–but the question is how to ensure a wise and proper application of MBE techniques [2, p. 123]. Although this is a highly relevant question, many other ethical and empirical concerns have emerged in the debate. In the following section, I identify three broad sets of concerns commonly raised in the context of MBE.

## Concerns regarding moral bioenhancement

More than a decade later, there is virtually no consensus about any aspect of enhancing morality by biomedical means. Difficulties include the disagreement about what counts as MBE (what constitutes morality and what it means to act morally) and the ethical assessment of permissibility and desirability of such interventions. Over time, the absence of consensus gave rise to various approaches to MBE, but the common denominator among critics is a significant amount of scepticism about its prospects.

In this section, I present and categorize the most persisting sets of concerns in the MBE debate. I identify three broad concerns: First, some worry that MBE is conceptually unsound or unfeasible, and that, even if it proves to be feasible, it may have undesirable effects. Second, some worry that even if MBE proves to be a coherent and feasible idea, it will still be morally impermissible because it threatens fundamental moral values like freedom and autonomy. Third, some worry that MBE will exacerbate existing socio-political problems such as inequality and injustice or cause new ones such as the attainment of higher moral status by the enhanced individuals.^5^

### Conceptuality and feasibility concerns

Like the broader discussion on human enhancement, there is a wide spectrum of approaches and enhancement categories regarding the types of capacities and the kinds of improvement in question. This diversity makes concerns of conceptuality and feasibility the most extensive category. Approaches diverge on what and how to enhance, what it means to enhance morally, and how to ensure wise and proper application. It is possible to distinguish between several types of capacities usually associated with improving moral judgment and behavior. On the one hand, we can try to improve affect, emotion, or motivation [10]. On the other hand, MBEs can be generally designated as “interventions that are intended to improve our moral capacities such as our capacities for sympathy and fairness” [11, p. 361]. These interventions could be implemented through three relevant kinds of improvement: (i) motivational, (ii) cognitive, and (iii) behavioral [11].

Similarly, some distinguish between behavioral, emotional, and dispositional biomedical moral enhancement [12]. Emotional enhancement, such as the one described previously by Persson and Savulescu, has been getting much attention across the debate. It directly changes the way we feel about specific behaviors (our emotions), as opposed to dispositional enhancement, which aims at reinforcing dispositions such as empathy and a sense of fairness; these dispositions ground the propensity to respond in a socially appropriate way in a specific context [12, p. 256]. This variety of approaches indicates how challenging it is to determine the conceptual and theoretical features of MBE.

Another issue is in understanding what it truly means to “morally enhance”? The target or subject matter here could mean making a better world; a morally better person; or improving the sphere of morality [13]. The second understanding is closest to what Persson and Savulescu had in mind, since it includes “making people more likely to act on their moral beliefs, improving their reflective and reasoning abilities as applied to moral issues, increasing their ability to be compassionate, and so on” [13, p. 253]. Conceptual puzzles translate into the domain of ethical evaluation in a sense that “competing concepts of morality and moral conduct…complicate a definition of moral enhancement and the assessment of suitable target features for moral enhancement measures to be applied” [14, p. 236]. Beck believes that rival (meta-)ethical theories have different views on what features should be targeted through enhancement; if we want to enhance moral conduct, we need to provide a unanimous answer [14, p. 234].

Depending on the preferred approach to MBE, it will be possible to differentiate between several kinds of MBE in practice. For example, one can categorize MBE in terms of the capacities that are being enhanced (such as affect, emotions, motivation, cognition, dispositions, etc.) or in terms of the techniques/interventions being used to arrive at MBE (such as pharmaceuticals, deep brain stimulation, genetic selection or engineering). Even if we manage to agree on the content of MBE, other difficulties of a practical nature will arise. For example, who would be responsible for making decisions about MBE? Should MBE be voluntary or mandatory? Should we target only certain groups–such as convicted criminals, morally corrupt individuals, and the mentally ill–or should we apply it widely to children, like vaccination? What would be the advantages and disadvantages?

Some believe that the absence of agreement on such a fundamental level indicates that MBE is not very likely to be made sense of or realized in the medium-term future [14]. However, while some do not see the prospect or need for MBE for the reasons given, others believe it is possible to reach an overlapping consensus about moral matters and to develop many kinds of effective MBEs [15]. Even if we are optimistic about the prospects of MBE, in terms of reaching an agreement on what to enhance and how, it remains to examine the moral and ethical permissibility of such interventions.

### A threat to moral values

If MBE turned out to be coherent and biotechnologically feasible, many concerns regarding its effects on valuable aspects of human life would be raised. Therefore, the most challenging philosophical question in the MBE debate is probably: Even if we had sufficient scientific knowledge and understanding of biology, neuroscience, and moral psychology to engage in this project, would MBE endanger some of the most valuable aspects of human life? It is often suggested that MBE will interfere with our freedom, identity, and autonomy by making our acts morally desirable, but automatic and unwitting. For example, John Harris famously argued that enhancing human morality by artificial means would undercut freedom of choice, the so-called “freedom to fall,” which many may find unacceptable regardless of the potentially good consequences [16].

Some may also believe that any compulsion (or absence of choice) in the moral domain directly undermines the praiseworthiness and blameworthiness of acts, as well as the agent’s moral responsibility. Along these lines, Jotterand argued that moral neuro-enhancement is not possible because we can only become better, morally improved people through the careful, reflective exercise of our moral agency, not through neural manipulation [17]. Another problematic aspect in this context is the fear of misfiring or causing moral decline. What if MBE has unintended side-effects–like destroying psychological tendencies that are actually good for us? What about diminishing the diversity of moral positions and eliminating healthy disagreement that is essential for moral progress?

Some scholars challenged the assumption that MBE necessarily means making people morally better [13], because even though moral enhancers might induce many benefits to be produced for us, they do not provide us with the truth about what is morally right [18]. Moreover, if moral enhancers only bring us to conform to the duties we think we have, things can go wrong (e.g., religious fanatics and terrorists often strongly believe their duties are moral). Also, greater altruism and empathy do not guarantee moral progress or doing the right thing because terrorists and suicide bombers can be altruistic and empathetic in devoting their lives to their cause [18, p. 340]. The example of the radical terrorist indicates that we can often judge the same action as moral or immoral, depending on the interpretation or moral system we adhere to [19]. Hence, MBE must take into account environing social contexts: “moral intuitions, virtues, and rules are not identical around the world; changing the social context can switch a classification of a moral enhancement into a moral deficit” [15, pp. 3–4]. Otherwise, MBE may undermine the existence of reasonable pluralism in modern liberal societies: “The question is not only whether moral enhancement might lead to only one moral judgment, but also whether moral enhancement might benefit some reasonable moral, philosophical, and religious doctrines over others” [20, p. 29].

A multitude of concerns outlined here indicates an apparent lack of common ground regarding the theoretical and normative foundations in the MBE debate. There is a tendency to believe that MBE will undermine various moral values, but it is not clear what values it would improve if applied, nor is there a consensus on what values ought to be improved. It is questionable whether a unanimous answer can be offered to this question since any answer by itself may threaten a healthy diversity. The issue with different individual standards of morality gradually translates into a broader discussion about social norms and, by extension, to the impacts of MBE on socio-political level.

### Socio-political concerns

The third set of concerns revolves around the social and political effects that the implementation of MBE could bring about. For example, some worry the science of bioenhancement might lead to arbitrary inequalities in access to political power and would, in effect, facilitate the unjust rule of authoritarians [21]. Also, MBE might represent a risk in terms of reinvigorating dangerous ideas about the extent of natural inequality in the possession of moral faculties [22]. A related concern is the possibility of abusing this technology in terms of potentially inducing free-riding when the virtuous could become exposed to the exploitation by the vicious [23]. This problem might also appear on the broader level in the form of domination or exploitation of enhanced societies by the unenhanced ones [24].

A matter closely related to that of social equality, concerns social justice. Some worry that MBE might not only foster abuse but also exacerbate injustice. For example, it is uncertain “whether the use of bioenhancements, especially by officials in political states, would create a more just world, or rather a less just world” [25, p. 347]. There may be a possibility of MBE exacerbating, rather than diminishing, existing social prejudices and distributive unfairness [25, p. 347]. It is often assumed that morally enhanced individuals would acquire some kind of higher moral status, which would then spiral into elitism and discrimination. For example, “the widespread use of moral enhancement would raise the standards for praise and blame worthiness, making it much harder for the unenhanced to perform praiseworthy actions or avoid performing blameworthy actions” [26, p. 501]. It seems that, in order for MBE to be successful and abuse-free, it would have to be undertaken collectively–applied on a global level. It is, however, doubtful that people will be willing to expose themselves to such interventions voluntarily on a massive scale. If the level of participation in society is inadequate (people are not willing to undergo MBE procedure voluntarily), we will face the problem of compulsory MBE, which would restrict or completely obliterate individual freedom [27].

In summary, MBE evokes various concerns on conceptual, normative and socio-political levels, and from various professional, individual, and societal perspectives. These challenges range from determining what and how to enhance, to ensuring proper and harmless application. There is no consensus about what it means to enhance morally or which standard of morality we should adhere to. Will MBE interventions threaten valuable aspects of human life? Is it likely that problems of social inequality and injustice will be exacerbated by MBE? These are some of the central questions in the discussion. Sadly, a satisfying or unanimous answer does not seem to be in sight. One attempt to respond to many of the listed concerns is the development of a more sophisticated account of MBE. In the following section, I present one type of MBE which, arguably, escapes many of these worries.

## Response to concerns: moral neuroenhancement?

Although we can use the term “moral neuroenhancement” (MNE) to cover a wide range of biomedical interventions used to improve morally relevant capacities, MNE also refers to a particular account of moral betterment.^6^ Proponents of this account rely strongly on neurotechnological findings and focus on a new set of tools that may foster moral improvement. These tools are broadly described as “neurotechnologies”. Neurotechnologies are meant to directly alter brain states and neural functions in such a way as to bring about desired moral improvement [28, p. 166]. All along, advances in neuroscience have been a promising ground for the development of enhancement technologies. There are many examples of neuroscientific research and findings that could be useful and relevant in “correcting” immoral behavior. These findings range from the administration of neurohormones such as oxytocin (which promotes pro-social attitudes like trust, sympathy, and generosity), to the manipulation of serotonin and testosterone levels (which can mitigate undue aggression, and increase fair-mindedness, willingness to cooperate, and the aversion to harming others). Brain stimulation devices have also proven useful in morally relevant areas by reducing impulsive tendencies in psychopaths, treating addiction, and improving self-control [28, p. 167]. Advocates of MNE are fully aware that such measures might be ethically and conceptually controversial, as well as morally impermissible, but in their opinion, this should not exclude the possibility of, nevertheless, using some moral neuroenhancers that have already been developed [28].

An *agential* conception of MNE may help us understand how moral betterment is to be achieved:

Moral neuroenhancement: Any change in a moral agent–effected or facilitated in some significant way by the application of a neurotechnology–that results, or is reasonably expected to result, in the agent being a morally better (i.e., more moral) agent. [28, p. 168]

They leave open the matter of what should count as moral “betterment,” indicating that this list might include various examples, such as “increased moral worth or praiseworthiness of the agent, increased moral excellence of the agent or increased moral desirability of the agent’s character traits” [28, p. 168]. Earp et al. complement the MNE account by developing the *functional-augmentative approach* to enhancement.^7^ Since they are aware that having “more” of some morally relevant function or capacity does not always constitute betterment, they argue that a morally adept agent should be able to “respond flexibly to different situations, and to employ or tap into different cognitive and emotional resources as necessary to arrive at the motives, decisions, and behaviors that are morally desirable given the context” [28, p. 169]. A narrow focus on boosting specific moral capacities will not do the job entirely (e.g., increased empathy can lead us astray when it comes to making certain moral judgments), so in order to produce better moral agents, we must distinguish between lower- and higher-order moral capacities. For example, *empathy* or a *sense of fairness* represent lower-order capacities–basic features of human psychology relevant for moral motivation and behavior. Second-order capacities are in fact *abilities* to know or identify when it is morally desirable to feel and act upon lower-order capacities:

So it wouldn’t be just “more empathy” (tout court) that would be expected to lead to the improvement of a moral agent, qua moral agent, but rather an increase in what might roughly be described as a kind of second-order empathic control–an ability to (1) know or to identify, whether consciously or unconsciously, when it is morally desirable to feel empathy and/or allow it to shape one’s outward behavior (and in what way), as well as (2) to be able to feel such empathy, or if necessary, suppress such feelings (or their effects on behavior), in accordance with (1). [28, p. 170]

In other words, enhancement of higher-order capacities would involve reflective, flexible, reasonable, and context-dependent modulation of moral responses and for this reason, it would be more reliable than the previously described approach of “blindly” increasing or diminishing specific moral capacities. The idea of higher-order MNE can be contrasted with Owen Schaefer’s [29] distinction between direct and indirect moral enhancement (ME). Schaefer argued that direct ME is problematic because it aims at bringing about particular ideas, motives, or behavior, making people commit to them without much room for rational deliberation. Indirect ME would, on the other hand, aim at enabling people to produce morally correct ideas, motives, and behavior without having them commit to their content [29, p. 261]. MNE resembles indirect ME in many aspects and it seems to be a solid *prima facie* case of ME.

Ideally, indirect ME (or similar techniques of MNE) could be resilient to many objections standardly raised against direct forms of ME, such as restriction of freedom or fear of unintended bad consequences. If indirect ME would, as Schaefer anticipates, improve the reasoning process itself without committing people to adopt specific beliefs, this would eliminate the threat to healthy disagreement essential for moral progress. It would also avoid potential problems of insufficient moral understanding (raised in the case of direct ME), as well as the problem of making morality “too easy” or “disconnected” from the world [3, 16]. If indirect means of ME would leave enough room for reflective reasoning, effort, and engagement, this would also reduce the potential threat of direct ME to authenticity, autonomy, and rational decision-making:

Since moral lessons, abilities, dispositions, etc., that are achieved or developed with the help of a neurotechnology–as opposed to directly caused by it (thereby preserving space for conscious reflection, effort, and engagement)–could be seen as posing less of a threat to such important issues as authenticity, autonomy, and rational deliberation.... [28, pp. 174–175]

Earp et al. envision “a *facilitating*, rather than *determining* role for any drug or neurotechnology” [28, p. 175]. This means that neuroenhancers should be administered as part of a richly contextualized process of moral learning, rather than in a vacuum.

Although MNE escapes many of the previously raised moral concerns, the overall state of the debate leaves many questions open. For example, even though indirect MNE appears conceptually more plausible, because it is meant to rest on less contentious higher-order notions of moral betterment, it is still overly speculative on empirical grounds. One may doubt whether sophisticated interventions such as MNE are at all feasible. Even if MNE would not endanger fundamental values like freedom and autonomy, its socio-political ramifications may still be concerning. These open concerns, together with lack of consensus and the debate’s continuous evolution, necessitate the devising of widely applicable methodological guidelines and evaluation criteria.^8^ More precisely, the debate on MBE, as it stands, is insufficiently theoretically informed. It seems to be caught up in details of fictional scenarios and implementations and their outcomes, whereas there is still fundamental disagreement at the conceptual and normative level. Although these ongoing discussions are valuable in their own right, we may benefit from ascending to a more general level of ethical analysis and by setting out a methodological framework for a systematic assessment of moral permissibility.^9^

## A methodological framework for the assessment of moral enhancement(s)

The previously detailed sets of concerns represent the toughest challenges in the MBE debate and, on the whole, are the best reasons in favor of MBE’s moral impermissibility. These concerns, however, may be the only common ground in the enhancement debate and roughly correspond to important theoretical and normative standards MBE should satisfy to, at least, *not* be considered downright unacceptable or impermissible. Accordingly, three general levels of requirements prove to be especially important across the debate on the permissibility of MBE techniques:

(i) plausible coherence, feasibility, and effectiveness of an MBE project;
(ii) the absence of conflict with fundamental moral values and norms;
(iii) compatibility with or facilitation of socio-political goals of equality and justice.

This is not to say that these requirements directly correspond to the above concerns or that satisfying these requirements would automatically guarantee moral permissibility. Instead, it means that exploring these conditions could give us a better understanding of what would or would not make MBE permissible.

In what follows, I explain the implementation of my approach in terms of ethical and theoretical analyses, and then give an example of how this approach can be applied, assuming that MBE becomes a possibility. The former also outlines why these criteria are needed, what questions they attempt to answer and how they are intended to be answered, and what contributions the investigation of these three perspectives would bring to the debate.

### Ethical and theoretical analyses

The first requirement entails determining whether MBE is (in some or all conceivable forms) a consistent and plausible proposal, and, in principle, feasible and effective. We want to answer here: Whether MBE would have the effects anticipated by its proponents? Which, in turn, requires asking: How does the proposal align with external but integrally linked theories (such as cognitive neuroscience, evolutionary psychology of emotions, etc.)? Are common assumptions made in the discussion warranted (such as that MBE is self-defeating)? In addition, this would also require addressing how potential problems ensuing from a close theoretical examination affect MBE’s moral permissibility. Establishing theoretical soundness and internal consistency of a particular MBE proposal can be achieved by tools such as thorough conceptual analysis, comparative analysis, and the application of available and relevant scientific data. If, for example, the theoretical analysis indicates that MBE by emotion modulation would misfire because emotions cannot reliably guide human moral decision-making, then this would impact our understanding of the proposal’s coherence and effectiveness, as well as its moral permissibility.^10^

Second, in order to assess whether MBE conflicts with fundamental moral norms and values, we must investigate its relationship with predominant moral views. Since normative ethics offers specific accounts of rightness and justifiability of particular acts and decisions, it should be an adequate point of departure to answer questions such as: Does MBE present a genuine threat to fundamental norms and values? Is MBE harmful? Can it produce good consequences or, perhaps, will it undermine intrinsically valuable things such as freedom, autonomy, and moral responsibility? Illuminating the matters of moral permissibility of MBE requires looking into particular accounts of moral rightness and permissibility if we want the assessment to be sufficiently theoretically informed.^11^ Therefore, the moral permissibility of MBE can be assessed through its accordance with the utilitarian principle of maximizing utility or the conformity with deontological rules.^12^

For example, the ongoing debate leaves a general impression that MBE would likely be justified on consequentialist grounds–as far as it maximizes the overall utility [35] and that it would be intrinsically (or deontologically) wrong as far as it undermines our freedom, autonomy, or responsibility [e.g., 16]. I believe these matters are worth digging into because they will, consequently, provide insight into whether MBE is, in fact, a threat to specific values and under which circumstances, if any, it could be acceptable.^13^

Finally, even if a particular MBE technique is morally justified (i.e., if it does not contradict prevailing norms), its social implications represent the next challenge. This requires examining the previously discussed puzzling questions and concerns that arise from MBE’s socio-political impacts. Some of those include answering: Whether MBE would deepen social inequality and injustice, or whether it could reduce the negative effects of the natural and social lottery? Whether MBE would be in an individual’s or in society’s interest? Would it provide grounds for exploitation and discrimination? Methods for tackling these concerns, in terms of conceptual and theoretical clarifications, would be the application of well-established theories of social justice and equality. For example, the Rawlsian maximin principle would require that any redistribution of social goods, say enhancements, benefits those who are the worst off.^14^ Hence, my third methodological guideline would provide a means for elucidating justice and equality matters in the enhancement debate and how they affect the overall moral permissibility of discussed technologies.

### Application of the criteria

Let me illustrate a preliminary application of the proposed criteria on a concrete, practical example.

Imagine Jane, who is inclined to become a lone wolf terrorist and intends to commit a terrorist act. Nevertheless, Jane decides to engage in MBE by emotion modulation–where she will have her altruism (empathy) increased and her sense of justice strengthened. Now, according to the first criterium, assessing whether this concrete MBE intervention is coherent, feasible, and effective, will, in practice, depend upon whether it will have the desired effects. The desired effects would involve Jane: (i) recognizing that her intentions (and, consequently, actions) are morally impermissible and (ii) refraining from committing the act of terrorism. By contrast, undesirable effects would involve MBE making Jane even more devoted to her cause, which would present the backfiring problem and render MBE ineffective (as it would not have the anticipated effects).

The assessment of MBE’s compatibility with fundamental moral values and norms (the second criterium) would, in practice, involve assessing whether Jane’s actions, *following* the enhancement intervention, are compatible with a set of widely accepted and uncontroversial moral norms and values. For example, let us imagine that after engaging in MBE, Jane decides to refrain from committing the terroristic act she previously intended. Since harming others is almost always morally wrong, Jane’s change of heart would be more than welcome. This is to say that Jane’s engagement in MBE would be morally right from, for example, consequentialist and deontological viewpoints. However, deontologists and virtue ethicists could further question whether her actions are praiseworthy or whether MBE has undermined her moral responsibility.

Finally, assessing whether MBE is compatible with or facilitates socio-political goals of equality and justice would require assessing the broader socio-political effects of Jane’s actions. Namely, Jane’s decision to engage in MBE and, by extension, to refrain from terrorism, is obviously a socially desirable endeavor because it aligns with principles of respect for fundamental human rights (e.g., the right to life and to not be harmed). Also, by not committing this crime, Jane would avoid incarceration, which is desirable individually and socially. However, if Jane would not engage in MBE voluntarily or if she would be stigmatized for engaging in it, some concerns about social justice and equality could be raised. For example, her individual rights could be infringed if MBE is coerced or if she is discriminated against for undergoing the enhancement intervention.

At this point we are facing the question of *how* to tackle various issues arising in Jane’s example, which leads us back to the previously described methodological suggestions for applying ethical analyses to the bioethical debate on MBE. Namely, assessing MBE interventions across the three presented categories (and implementing the previously described methods) should provide conclusions about its theoretical soundness and feasibility, compatibility with fundamental moral norms, and compatibility with or facilitation of socio-political goals. On the whole, it should facilitate our understanding of what is or is not permissible.

## Conclusion

This article narrowed down the list of factors that may be crucial for elucidating what may or may not be morally permissible in the debate on biomedical moral enhancement. To identify these relevant factors, I reviewed the debate and showed that it requires a more rigorous ethical analysis. A lack of consensus about enhancements’ desirable features and the constant debate on development call for clear and precise methodological guidelines. I proposed three important theoretical and normative standards that MBE should satisfy if we wish to mitigate the concerns about its utter impermissibility. These include (i) plausible coherence, feasibility, and effectiveness of the MBE project; (ii) absence of conflict with fundamental moral values and norms; and (iii) compatibility with or facilitation of socio-political goals of equality and justice. Exploring how MBE corresponds to these requirements will give us a better understanding of whether and under what conditions it is permissible, or at least what would make it permissible. It will also provide a theoretical and normative basis for a more fruitful discussion.

## Data Availability

Not applicable.

## Footnotes

1 For simplicity, I will henceforth use the term moral bioenhancement (MBE) to refer to a variety of biomedically induced moral enhancements, as opposed to non-biomedical moral enhancements such as upbringing, socialization, etc.).

2 This work is an extension and application of the same methodological framework developed in [7].

3 The term ‘groupishness’ originates from Jonathan Haidt’s book *The righteous mind: why good people are divided by politics and religion* [9]. This stands for the psychological phenomenon of group or social selfishness–and it manifests in selfish motivations such as loyalty, which promote survival and interests of the group one belongs to.

4 Just because something is grounded in biology does not necessarily mean it can be modified by biomedical means. The intention here is to say that, all things considered, this may be possible *in principle* for MBE.

5 This is not to suggest that these levels exhaust all possible ethical implications of MBE. However, they seem to adequately cover a wide range of pertinent issues raised in the MBE debate, and thus require closer examination.

6 Please note that here, MNE refers to a more theoretically sophisticated account of MBE, but practically speaking, MNE and MBE belong to the same category of enhancements.

7 This approach aims to show that “[i]nterventions are considered enhancements … insofar as they [augment] some capacity or function (such as cognition, vision, hearing, alertness) by *increasing the ability of the function to do what it normally does*” [28, p. 168].

8 For example, one of the latest developments in the debate has been a proposal to shift from MBE to AI-based moral enhancement by introducing an “artificial moral advisor” [e.g., 30, 31]. Virtual reality could also play a role in enhancing human empathy [32]. I believe the conclusions reached in this paper would apply to such “digitized” versions of MBE, too.

9 I have also developed and proposed this methodological intervention at the human enhancement level in [7]. Here I want to make use of it on a more concrete example of moral enhancement.

10 I discuss the role of emotions in the MBE debate in more detail in [33].

11 There have been attempts to discuss enhancements from different normative perspectives, but this is rarely done explicitly and systematically. For example, the welfarist account [34] is an inherently normative approach instructing us to enhance whenever the intervention increases overall wellbeing, but it does not provide us with clear criteria or content for how to understand and evaluate well-being. These gaps can be filled by applying familiar normative models that give more straightforward instruction on discerning permissible from impermissible acts.

12 This is not to say that we should base MBE on preferred ethical theories, but rather that we should use ethical theories to inform and illuminate the question of moral permissibility in the enhancement debate.

13 I discuss in detail whether MBE is justified on utilitarian grounds in [36] and outline some conditions and constraints on how and why we might engage in MBE on Kantian grounds in [37]. Some scholars have also mapped a number of consequentialist arguments in the human enhancement debate [38], while others discussed the potential negative effects of MBE on utilitarianism [5]. Deontological analysis of moral enhancement has also been receiving some attention, (see, e.g., [39]); but more has been said about human enhancement in this regard, (see, e.g., [40–42]). Most of these studies indicate that enhancements would not necessarily undermine deontological values, as we may have originally thought.

14 This approach mirrors the previously discussed application of normative theories to the matters of moral permissibility of MBE. After all, theories of social justice and equality are to some extent a subset of normative theories. I thank the anonymous reviewer for leading me to this point.
